# Symmetrized Dot Patterns and CNN-Based Acoustic Signal Analysis for Fault Diagnosis in Internal Combustion Engines

**DOI:** 10.3390/s26154873

**Published:** 2026-08-02

**Authors:** Robinson Xavier Rojas Espinoza, Rafael Wilmer Contreras Urgiles, Milton Garcia Tobar

**Affiliations:** Grupo de Investigación en Ingeniería del Transporte, Universidad Politécnica Salesiana, Cuenca 010105, Ecuador; rrojase@est.ups.edu.ec (R.X.R.E.); rcontreras@ups.edu.ec (R.W.C.U.)

**Keywords:** internal combustion engines, acoustic signal analysis, Symmetrized Dot Patterns, convolutional neural networks, fault detection, MEMS microphones

## Abstract

Early detection of faults in internal combustion engines (ICEs) remains an important challenge for improving operational reliability and supporting condition assessment. Conventional approaches based on On-Board Diagnostics II OBD-II data, vibration, or exhaust gas analysis face practical limitations, motivating the exploration of non-invasive acoustic diagnostics. This study proposes and validates a pipeline that combines acoustic signals, Symmetrized Dot Pattern (SDP) transformations, and convolutional neural networks (CNNs) to identify injector and ignition failures in multicylinder ICEs. Acoustic measurements were acquired in a purpose-built semi-anechoic chamber using a smartphone equipped with Micro-Electro-Mechanical Systems (MEMS) microphones, ensuring high-signal-to-noise-ratio recordings under controlled fault conditions. A total of 540 independent acoustic recordings were transformed into 540 grayscale SDP images, which were used to train and test a custom CNN architecture. Results demonstrated an overall accuracy of 81.11% in independent verification, with outstanding performance in Spark Plug 3 and Spark Plug 4 (F1 = 1.000), as well as Injector 4 and normal operation (F1 = 0.947). More challenging cases included Injector 1, Injector 3, and Spark Plug 1 (F1 ≈ 0.70–0.78), where spectral overlap led to cross-misclassifications. These findings are consistent with recent studies reporting the robustness of SDP for representing transient acoustic phenomena and the superior classification capability of CNNs for pattern recognition tasks. The study extends SDP applications to internal combustion engines, demonstrating the feasibility of detecting injection and ignition faults using acoustic measurements. The proposed SDP–CNN pipeline represents a non-invasive, low-cost approach to acoustic fault analysis using consumer-grade sensing devices. Future research should focus on expanding datasets, incorporating domain adaptation techniques, and validating performance under real-world operating conditions to improve generalization and support practical acoustic monitoring applications.

## 1. Introduction

Early fault detection in internal combustion engines (ICEs) remains an important challenge for ensuring reliable operation and maintaining adequate combustion performance [1]. Failures in injection or ignition systems, such as faulty injectors or degraded spark plugs, can alter the combustion process and generate detectable changes in engine operating conditions [2,3].

Traditional diagnostic approaches have primarily relied on information from electronic control units via on-board diagnostic (OBD-II) systems [4], vibration analysis, and exhaust gas analysis [4,5,6]. Although effective in specific contexts, these methods have several limitations: OBD-II access is not always available, vibration-analysis instrumentation requires specialized installation, and exhaust-gas analyzers are costly and impractical for field applications [7,8].

As an alternative, non-invasive acoustic methods have emerged as a promising option for external engine monitoring using low-cost microphones. The main challenge lies in the high variability of acoustic signals, which are strongly influenced by ambient noise and the engine’s dynamic operating conditions [9,10]. Previous studies have also demonstrated that acoustic signals acquired using smartphones equipped with Micro-Electro-Mechanical Systems (MEMS) microphones can provide diagnostically relevant information for fault detection in internal combustion engines when combined with advanced signal processing techniques [11,12]. To address this, alternative signal representations have been explored to more robustly capture temporal and spectral characteristics, including spectrograms, wavelet transforms, and, more recently, the Symmetrized Dot Pattern (SDP) [13,14,15].

The SDP method transforms one-dimensional sequences into two-dimensional polar distributions that simultaneously preserve amplitude distribution and temporal structure, offering advantages over conventional representations. Its main benefits include robustness against background noise, increased sensitivity to short-duration transient phenomena, and improved capability to distinguish between normal and abnormal states, even under low-speed operating conditions [16]. Furthermore, by converting signals into images, SDP can be naturally integrated with convolutional neural networks (CNNs), enabling automatic feature extraction and reducing dependence on manual feature engineering [17].

Recent studies have demonstrated the effectiveness of combining SDP with deep learning in highly complex scenarios. Wu et al. demonstrated its applicability for acoustic classification [13], while Yin et al. validated its robustness in diagnosing composite faults in rotating systems, even under noisy conditions and multiple excitation sources [17]. Similarly, Tang et al. showed that feature selection applied to SDP images significantly improves diagnostic accuracy in rotating machinery [16]. Nevertheless, despite these advances, studies integrating acoustic signals, SDP representations, and convolutional neural networks for fault detection in multicylinder internal combustion engines remain limited, representing a relevant gap in the current literature.

The adoption of machine learning and deep learning techniques has significantly transformed fault diagnosis in internal combustion engines. Models such as artificial neural networks (ANNs), convolutional neural networks (CNNs), deep neural networks (DNNs), and probabilistic neural networks (PNNs) have demonstrated competitive performance in fault classification and detection tasks [18,19,20,21,22]. These methodologies have been successfully applied to vibration signals, acoustic emissions, and sensor-based measurements, achieving accuracy levels exceeding 80% in scenarios involving multiple fault conditions [2].

In this context, the present study proposes an acoustic signal analysis methodology based on Symmetrized Dot Patterns and convolutional neural networks to detect injector- and spark plug-related faults in multicylinder internal combustion engines. Measurements were performed in a semi-anechoic chamber specifically designed for engine acoustic diagnostics, enabling the acquisition of high-quality signals under controlled conditions. The proposed methodology integrates signal acquisition, spectral and visual transformation through SDP, and automated classification using CNNs.

The remainder of this paper is organized as follows. Section 2 describes the experimental setup, signal preprocessing procedures, and the classification model employed. Section 3 presents the training, validation, and verification results, including class-wise performance metrics. Section 4 discusses the implications of the findings for acoustic fault detection and automated pattern recognition in internal combustion engines. Finally, Section 5 summarizes the main conclusions and outlines future research directions.

## 2. Materials and Methods

This section describes the methodological and technical procedures employed in this study. It begins with the research methodology, followed by a description of the experimental setup used to acquire acoustic data under controlled conditions. The subsequent sections present the signal-processing workflow that transforms acoustic signals into Symmetrized Dot Pattern (SDP) images via Discrete Fourier Transform (DFT)-based spectral analysis. Finally, the architecture and training procedure of the convolutional neural network (CNN) implemented in MATLAB R2024a are detailed, emphasizing the process for classifying engine operating conditions based on visual features extracted from SDP representations.

### 2.1. Research Methodology

**Figure 1:**
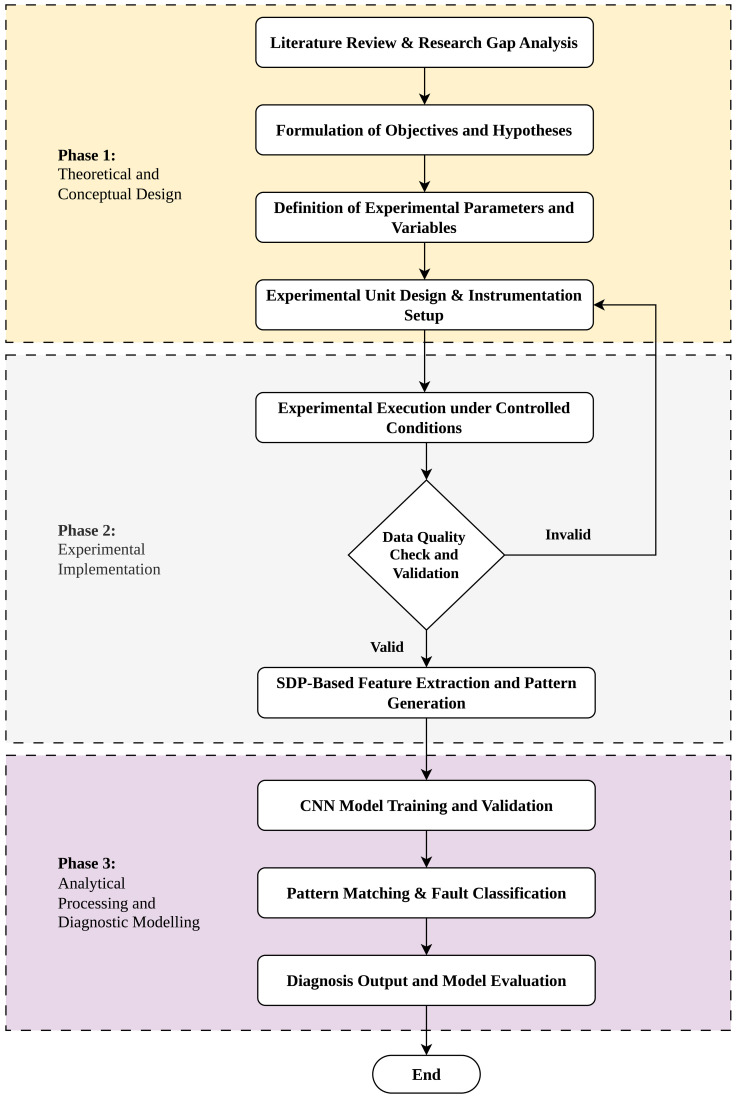
Flowchart of the methodological workflow adopted in this study.

The research methodology adopted in this study follows a structured experimental and computational workflow that integrates signal acquisition, spectral transformation, image-based pattern representation, and machine-learning classification. As illustrated in Figure 1, the methodological sequence was organized into three main stages to ensure consistency among physical measurements, data processing, and classification outcomes.

In the first phase, a conceptual and theoretical foundation was established through a review of the high-impact scientific literature on non-invasive acoustic analysis and the Symmetrized Dot Pattern (SDP) method. This review guided the definition of key experimental variables and informed the development of algorithms for transforming time-domain acoustic signals into visual representations suitable for pattern recognition. This stage employed an inductive approach to identify the parameters that most significantly influence the quality and discriminative capability of SDP images.

In the second phase, the experimental protocol was implemented using a four-cylinder internal combustion engine instrumented to simulate controlled fault conditions. Acoustic signals were recorded under steady-state operation across predefined scenarios, including injector and ignition faults. This phase combined empirical data acquisition with systematic signal-processing procedures to generate grayscale SDP images. The resulting images were evaluated for consistency and noise suppression, with support from the semi-anechoic environment and standardized microphone positioning.

In the third phase, the classification task was addressed. Convolutional neural networks (CNNs) were trained using the generated dataset to learn discriminative features associated with each operating condition. The CNN was optimized through iterative training, validation, and hyperparameter adjustment. The model was subsequently evaluated using previously unseen data to assess classification performance. This phase completed the methodological workflow, transforming raw acoustic measurements into automatically classified engine operating conditions.

### 2.2. Experimental Unit

The experimental setup used in this study is based on a four-cylinder gasoline internal combustion engine. The engine is equipped with a dedicated diagnostic control panel that enables the operator to safely and repeatedly simulate various fault conditions. The panel incorporates a set of switches that allow the selective disconnection of injectors or spark plugs without requiring direct physical intervention. This capability ensures consistent fault induction and enhances the repeatability of the experimental procedure. The technical specifications of the engine are summarized in Table 1.

Acoustic signal acquisition was carried out using an iPhone 15 smartphone (Apple Inc., Cupertino, CA, USA) equipped with an integrated MEMS (Micro-Electro-Mechanical Systems) microphone array. The device incorporates three internal digital microphones commonly used for high-fidelity audio recording and active noise cancellation. For this study, the primary microphone was oriented toward the engine and used as the acoustic sensor.

Previous studies have demonstrated the feasibility of using smartphones for fault detection in internal combustion engines, highlighting their ability to capture diagnostically relevant acoustic information when combined with advanced signal processing techniques [11,12,23]. In the present work, the smartphone’s sampling frequency and integrated MEMS technology provided suitable characteristics for time–frequency analysis based on the Symmetrized Dot Pattern (SDP) method.

All experiments were conducted inside a semi-anechoic chamber specifically designed for engine acoustic measurements, ensuring a high signal-to-noise ratio and consistent acquisition conditions. This environment attenuates environmental and reflected noise, enabling clean signal acquisition and reproducible experimental results.

**Figure 2:**
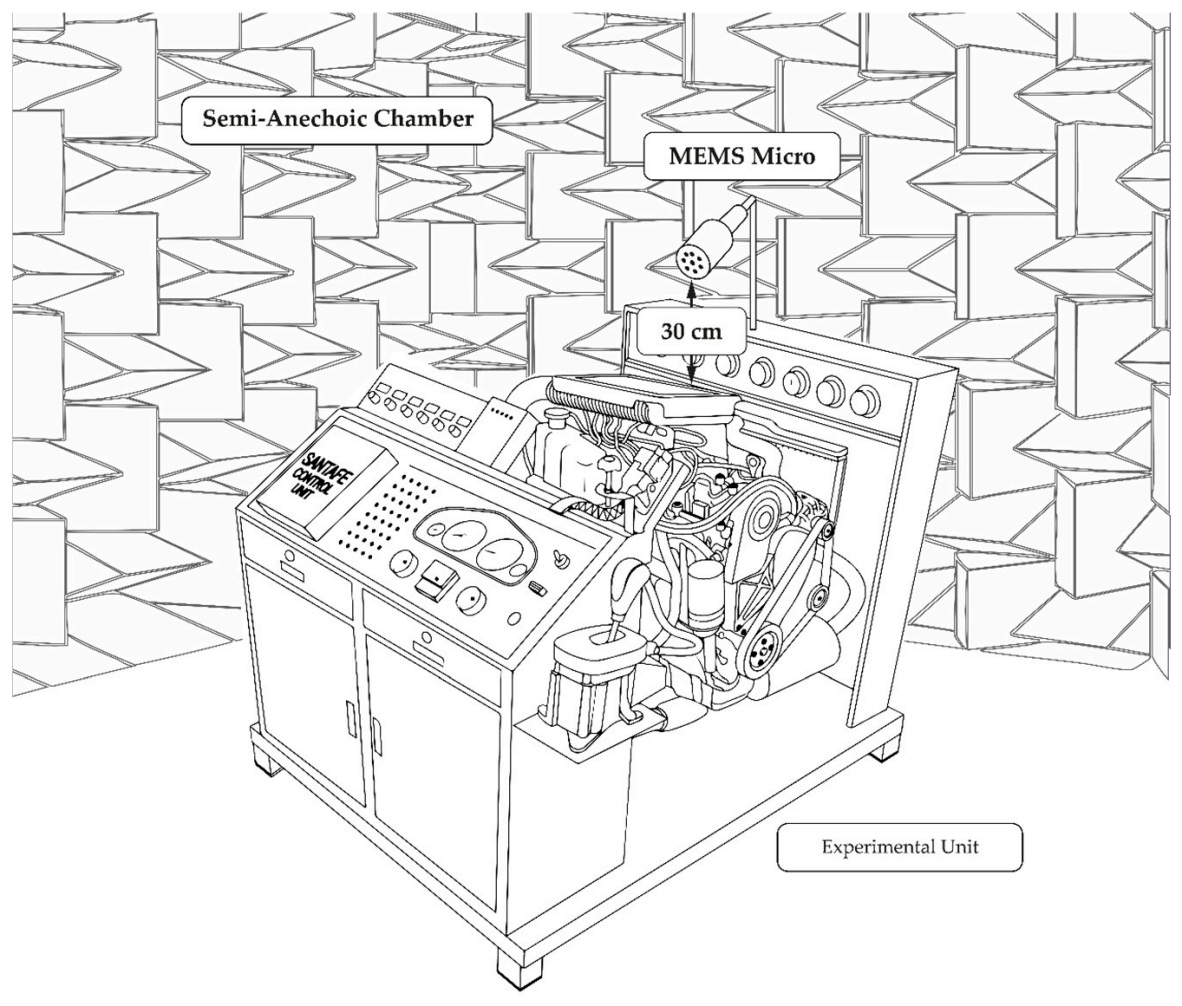
Experimental setup for acoustic signal acquisition in a semi-anechoic chamber.

Microphone positioning followed the recommendations of ISO 3745:2012 [24]. The device was placed at a height of 30 cm, with the radiator cap as the spatial reference point, and inclined at 45° relative to the horizontal plane. This configuration standardized the acoustic path and facilitated comparative analysis among the different test scenarios. The setup is illustrated in Figure 2.

The technical characteristics of the smartphone microphone system are summarized in Table 2.

### 2.3. Data Acquisition

The acoustic data were recorded under steady-state conditions, with the engine operating at idle and the throttle held at a constant position. The engine operated at an idle speed of 850 rpm. All measurements were conducted using Ecopaís gasoline (regular-grade, ethanol-blended fuel commercially available in Ecuador) at an ambient temperature of 22 °C. Each measurement was performed after the engine reached thermal stability, with the coolant temperature ranging from 90 °C to 98 °C, ensuring consistent operating conditions across all repetitions. To eliminate external acoustic disturbances, the electric cooling fan was disabled during the recording process.

Nine operating conditions were considered in this study: normal operation, individual injector deactivation for cylinders 1 through 4, and individual spark plug faults for cylinders 1 through 4. In ignition-fault scenarios, the spark plug electrode gap was set to zero to disable ignition in the corresponding cylinder while maintaining normal operation in the remaining cylinders. This procedure enabled the emulation of misfire conditions without introducing additional mechanical disturbances.

For each operating condition, sixty independent acoustic recordings were collected, resulting in a total dataset of 540 audio samples. Each recording lasted 10 s and was acquired at a sampling frequency of 48 kHz with 16-bit resolution. Each recording was subsequently converted into a single Symmetrized Dot Pattern (SDP) image, establishing a one-to-one correspondence between the acquired acoustic recordings and the generated dataset. The acoustic data were stored in uncompressed WAV format to preserve signal integrity and facilitate subsequent processing. All recordings were obtained using the previously described setup inside a semi-anechoic chamber, minimizing environmental reflections and ensuring high-quality signal acquisition.

Signal acquisition followed a standardized protocol. Each session began after engine stabilization and was followed by activation of the diagnostic control panel to simulate the target fault condition. The microphone remained fixed in both position and orientation throughout all experiments to ensure geometrical and acoustic consistency. Each recording was manually reviewed to verify the absence of transient noise or acquisition artifacts. No additional filtering was applied, as the semi-anechoic environment provided sufficient isolation for clean waveform acquisition.

Validated audio signals were incorporated into a structured database and used as input for subsequent analysis. The raw waveforms were transformed into Symmetrized Dot Pattern (SDP) images following the procedure described in Section 2.4. These visual representations constituted the input dataset used for fault classification through convolutional neural network (CNN) models.

### 2.4. Algorithm for Obtaining Dot Patterns

The Symmetrized Dot Pattern (SDP) technique was applied to extract representative features from the acoustic recordings. This method transforms one-dimensional temporal signals into two-dimensional polar visual patterns while preserving the signal’s amplitude distribution and structural characteristics. These visual representations are particularly well-suited to pattern recognition tasks with convolutional neural networks (CNNs).

The processing workflow begins with acquiring the raw acoustic signal in WAV format. Each recording is transformed into the frequency domain using the Discrete Fourier Transform (DFT), implemented through the Fast Fourier Transform (FFT). This transformation reveals the signal’s spectral content and serves as input to the subsequent SDP mapping. The DFT is mathematically expressed as Equation (1):

(1) $$X(k)=\sum_{n=0}^{N-1}x\left(n\right)\cdot e^{-j\cdot \frac{2\pi }{N}\cdot kn},\;k=0,1,\ldots ,N-1$$

where x(n) denotes the discrete-time acoustic signal, N is the total number of samples, and X(k) is the complex spectral value at frequency bin k. Only the magnitude spectrum obtained from the DFT was used to generate the SDP representations, whereas the phase information was intentionally omitted. Although phase information is not explicitly preserved, the magnitude spectrum retains the dominant energy distribution associated with each engine operating condition, providing a stable and discriminative representation for subsequent image-based classification. This approach follows the conventional SDP formulation reported in the literature and facilitates the extraction of robust spatial features by convolutional neural networks.

Once the magnitude spectrum is obtained, the SDP transformation maps each spectral component into a polar coordinate system. The radial coordinate and the two symmetric angular coordinates are defined by Equations (2)–(4):

(2) $$r\left(i\right)=\frac{x_{i}-x_{min}}{x_{max}-x_{min}},$$

(3) $$\theta \left(i\right)=\theta +\frac{x_{i+l}-x_{min}}{x_{max}-x_{min}}\xi ,$$

(4) $$\varphi \left(i\right)=\theta +\frac{x_{i+l}+x_{i+2l}-x_{min}}{x_{max}-x_{min}}\xi ,$$

where $x_{i}$ is the amplitude of the *i*-th spectral component, $x_{min}$ and $x_{max}$ are the minimum and maximum values in the spectrum, θ is the base angle, ξ is the angular amplification facto, l is the radial length parameter, and N denotes the total number of spectral samples obtained from the DFT.

To ensure numerical consistency during the SDP transformation, the index i was restricted to the valid spectral range satisfying i+2l≤N. This constraint guarantees that all shifted spectral components required by Equations (3) and (4) remain within the available spectrum, thereby preventing boundary effects during image generation. Consequently, no zero-padding, circular indexing, or extrapolation procedures were applied, preserving the integrity of the original spectral information throughout the SDP construction process.

To justify the selection of the Symmetrized Dot Pattern (SDP) parameters, a sensitivity analysis was conducted by evaluating the following representative combinations of the radial length parameter *l* (20, 80, and 100) and the angular amplification factor ξ (15°, 30°, and 45°), while maintaining all other experimental conditions unchanged.

**Figure 3:**
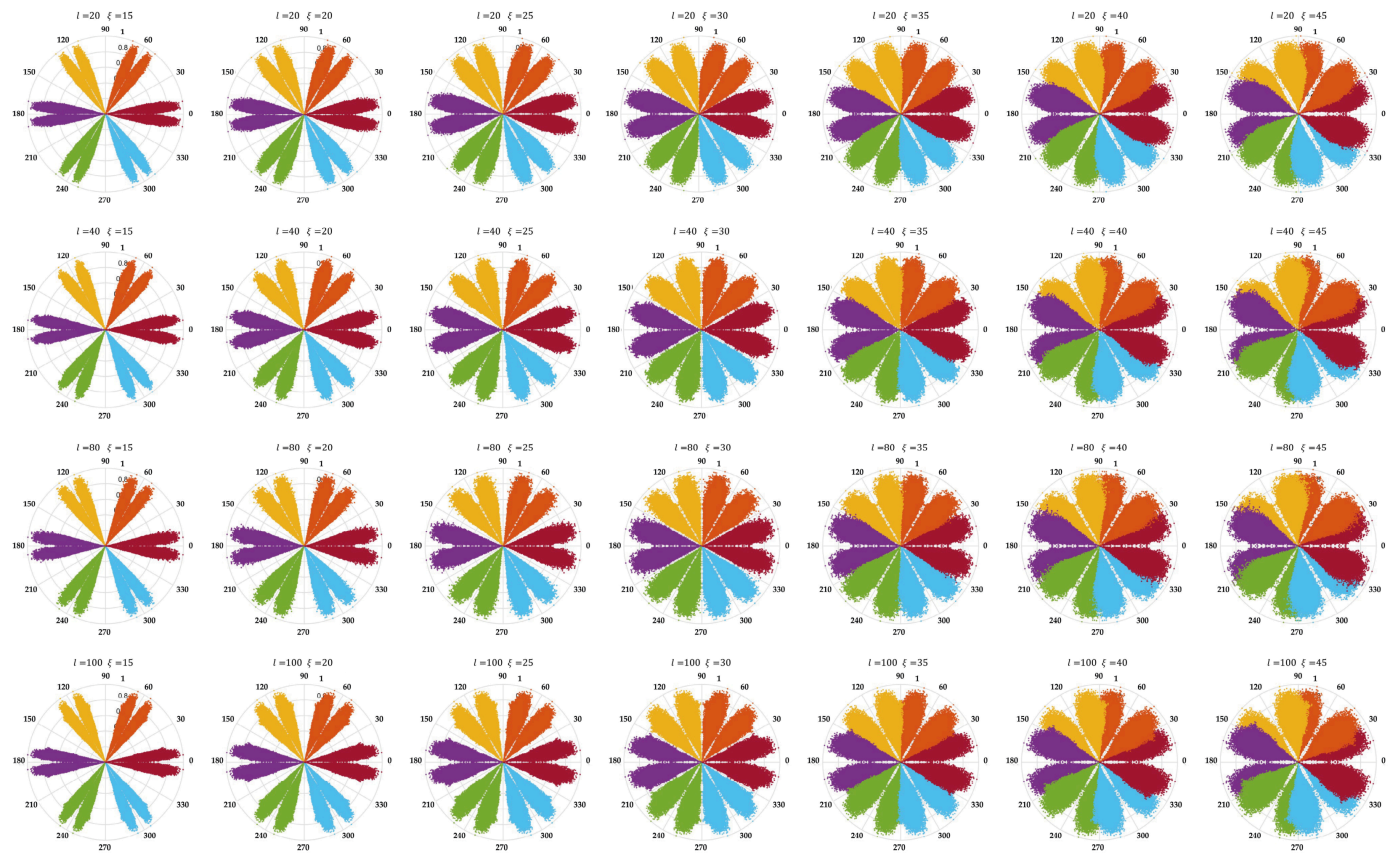
Analysis of the SDP parameters obtained by varying the radial length parameter (l) and the angular amplification factor (ξ).

Figure 3 illustrates the influence of both parameters on the geometric characteristics of the resulting SDP representations. As the radial length parameter increased, the radial structures became more elongated and the separation between lobes improved. Likewise, the angular amplification factor directly controlled the angular spacing between adjacent lobes, substantially influencing the visual discriminability of the generated patterns.

As illustrated in Figure 3, smaller values of l produced short radial structures with reduced lobe separation, limiting the representation of the spectral distribution. Similarly, low values of ξ generated compact patterns with insufficient angular separation, making the acoustic signatures less distinguishable.

Conversely, increasing ξ beyond $35^{\circ }$ progressively enlarged the angular spacing between adjacent points, resulting in excessive dispersion and partial overlap of the lobes. Likewise, very large values of l(100) generated elongated structures without providing additional visual discrimination.

To complement this qualitative assessment with quantitative evidence, each parameter combination was used to generate an independent SDP dataset. Table 3 summarizes the resulting classification accuracy for each evaluated parameter combination. These datasets were subsequently evaluated using the same CNN architecture and identical training hyperparameters described in Section 2.5, while adopting a preliminary 70/30 partition, of the 450-image development dataset, independent of the 80/20 partition employed for the final optimized model. This preliminary analysis was performed exclusively to support parameter selection and should not be interpreted as the final model evaluation presented in Section 3.

Among all evaluated combinations, l = 80 and ξ = 30° achieved the highest classification accuracy, consistent with the qualitative assessment shown in Figure 3, and were therefore adopted for generating all SDP images used throughout the remainder of this study.

**Figure 4:**
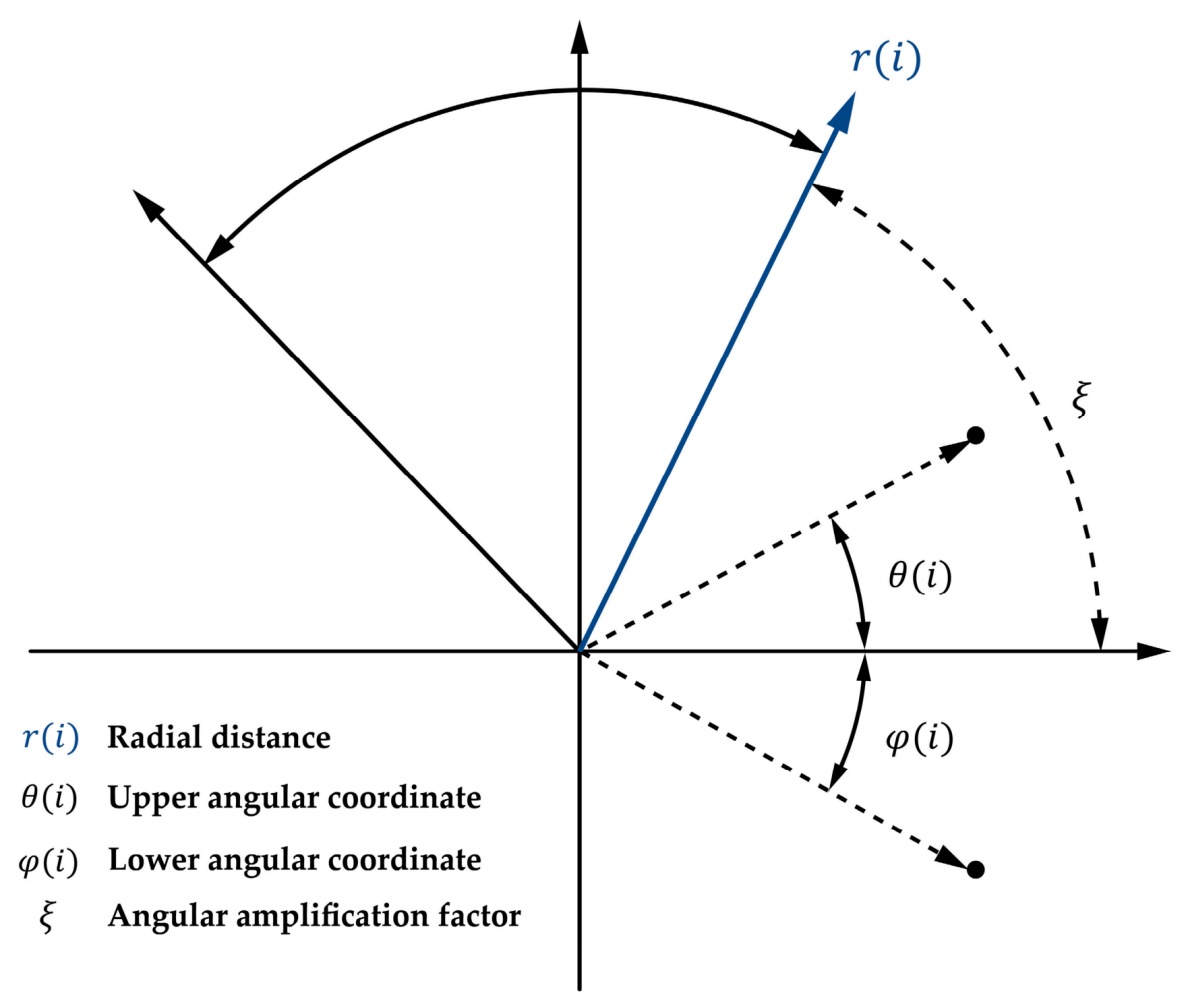
Variables in polar plane.

Figure 4 illustrates the geometric interpretation of these variables within the polar coordinate system. Each magnitude point and its symmetric reflection are plotted to generate the final dot pattern representing the acoustic signature associated with a given engine operating condition.

Each magnitude point and its symmetric reflection are plotted to generate the final dot pattern representing the acoustic signature of a given engine operating condition. The resulting patterns are stored as grayscale PNG images with a resolution of 224 × 224 pixels. These images constitute the input dataset for the CNN classifier described in Section 2.5.

**Figure 5:**
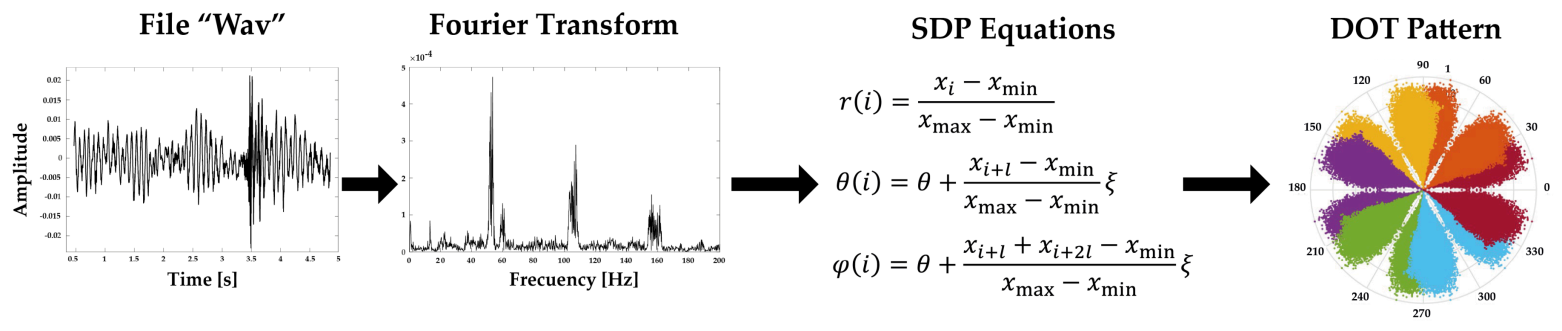
Workflow for generating Symmetrized Dot Pattern (SDP) images.

Figure 5 summarizes the complete transformation process from the raw acoustic waveform to the SDP image, including spectral transformation, polar mapping, and image generation.

Because the resulting patterns are not readily distinguishable through visual inspection, convolutional neural networks were employed for automated classification. The resulting dataset consisted of 540 SDP images, which constituted the input for the CNN classifier described in the following section.

The first subset consisted of 50 SDP images per operating condition (450 images in total) and was exclusively used for CNN development. These images were employed for model training and validation during the architecture optimization process.

The second subset consisted of the remaining 10 SDP images per operating condition (90 images in total). These images were completely excluded from the model development stage and were reserved exclusively for the final independent verification of the trained CNN. Consequently, none of these images participated in network training, validation, hyperparameter tuning, or architecture selection.

**Figure 6:**
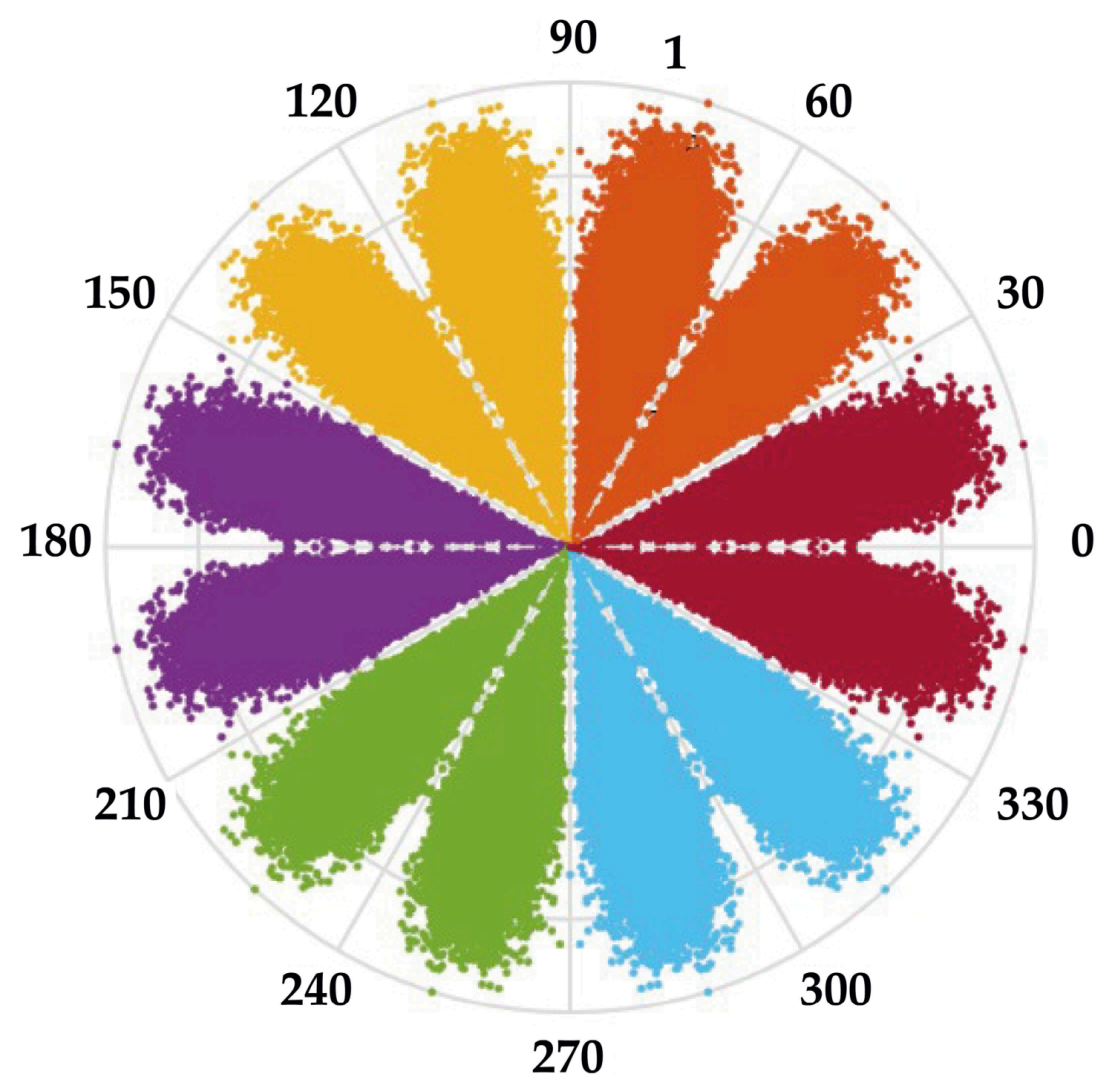
SDP pattern corresponding to normal engine operation.

**Figure 7:**
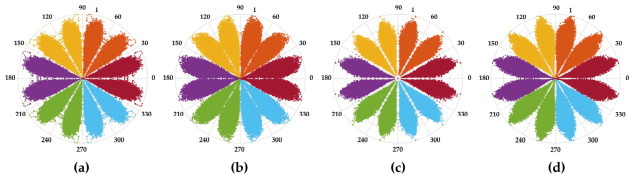
SDP patterns obtained under injector fault conditions: (**a**) Injector 1, (**b**) Injector 2, (**c**) Injector 3, and (**d**) Injector 4.

**Figure 8:**
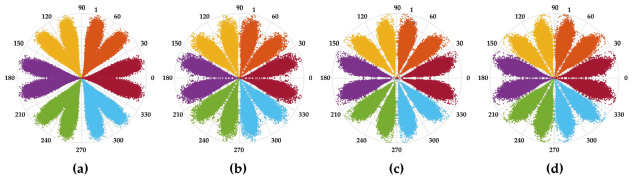
SDP patterns obtained under spark plug fault conditions: (**a**) Spark Plug 1, (**b**) Spark Plug 2, (**c**) Spark Plug 3, and (**d**) Spark Plug 4.

As illustrated in Figure 6, Figure 7 and Figure 8, the SDP transformation generated distinctive visual patterns associated with different engine operating conditions. Figure 6 presents the pattern corresponding to normal engine operation, which served as the reference condition throughout the study.

Figure 7 and Figure 8 show the SDP patterns obtained under injector and spark plug fault conditions, respectively. Although the visual differences among the generated patterns are subtle, the SDP representation preserves the underlying spectral structures that deep learning algorithms can exploit for automatic classification.

### 2.5. Classification Model

Given the subtle geometric differences among the Symmetrized Dot Pattern (SDP) images corresponding to the nine engine operating conditions, a Convolutional Neural Network (CNN) was adopted to automatically extract hierarchical spatial features and perform multiclass classification.

The CNN was implemented in MATLAB R2024a using the Deep Learning Toolbox. All SDP images were resized to 224 × 224 pixels with three color channels (RGB) before being introduced into the network. The implemented architecture consisted of an image input layer followed by 12 convolutional layers. Each convolutional layer was immediately followed by batch normalization and a Rectified Linear Unit (ReLU) activation function to improve convergence stability and facilitate gradient propagation during training. The convolutional filters progressively increased from 16 to 126, enabling the network to learn increasingly discriminative spatial representations of the SDP images.

Unlike conventional image-classification networks that primarily use 3 × 3 kernels, most convolutional layers in the proposed architecture use 6 × 6 kernels, with one intermediate layer using 3 × 3 kernels. The use of larger kernels was motivated by the geometric characteristics of SDP images, which contain broad, symmetric radial structures rather than fine local textures. Consequently, larger receptive fields facilitate the extraction of global spatial relationships among the radial lobes generated by the SDP transformation.

To progressively reduce the spatial dimensions while preserving the most representative information, seven max-pooling layers were incorporated, with 3 × 3 and 2 × 2 pooling windows, depending on the stage of the architecture.

Following feature extraction, the learned representations were processed through five fully connected layers containing 256, 128, 64, 32, and 9 neurons, respectively. The first four fully connected layers employed ReLU activation functions, whereas the final fully connected layer produced the probabilities for the nine operating conditions via a Softmax layer, followed by a multiclass classification layer with categorical cross-entropy loss.

The complete SDP database consisted of 540 images, corresponding to 60 SDP images for each of the nine engine operating conditions. To ensure an unbiased evaluation of the proposed methodology, the dataset was divided into two completely independent subsets. The model-development dataset consisted of 50 SDP images per operating condition (450 images). These images were exclusively used during CNN development and were randomly divided into 80% for training (360 images) and 20% for validation (90 images).

The remaining 10 SDP images per operating condition (90 images) were reserved for external verification. These images were never used during network training, validation, hyperparameter adjustment, or architecture selection. They were introduced only after the final CNN configuration had been fully trained to independently evaluate the classification capability of the proposed methodology.

Training was performed using the Adam optimizer with an initial learning rate of 0.001, a maximum of 60 epochs, random shuffling before every epoch, and a validation frequency of 80 iterations. Batch normalization was employed after every convolutional layer to improve optimization stability. No dropout layers or explicit L2 weight regularization were incorporated into the implemented architecture.

**Figure 9:**
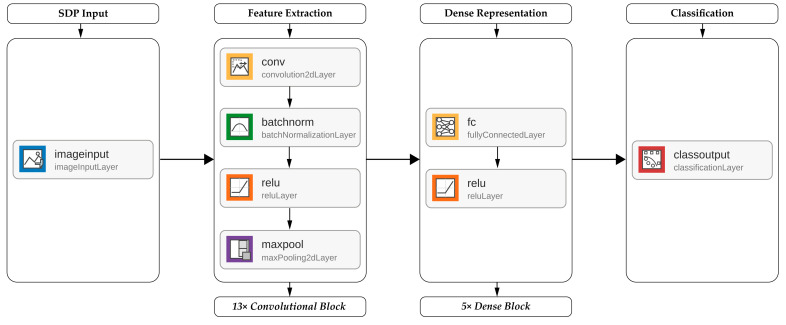
Conceptual workflow of the proposed CNN classifier.

The complete CNN architecture and the adopted training hyperparameters are summarized in Table 4 and Table 5, respectively, whereas Figure 9 illustrates the overall processing pipeline of the proposed CNN classifier. Considering the relatively limited dataset size, the proposed model should be regarded as a proof of concept developed under controlled laboratory conditions. Future studies should investigate lighter CNN architectures, larger databases, additional operating conditions, and cross-engine validation to improve the generalization capability of the proposed approach.

The proposed CNN architecture comprised approximately 2.4 million trainable parameters distributed across twelve convolutional layers and five fully connected layers.

Batch normalization was incorporated after each convolutional layer to improve optimization stability, accelerate convergence, and mitigate internal covariate shift during training. Preliminary experiments indicated that the combined use of batch normalization, progressive max-pooling, and hyperparameter optimization provided satisfactory convergence and validation performance under the adopted training configuration. Consequently, neither dropout layers nor explicit L2 weight regularization were incorporated into the final architecture. The proposed CNN should therefore be regarded as a proof-of-concept model intended to evaluate the feasibility of SDP-based acoustic representations under controlled laboratory conditions rather than as a fully optimized architecture for large-scale deployment.

## 3. Results

### 3.1. Training and Validation Performance

**Figure 10:**
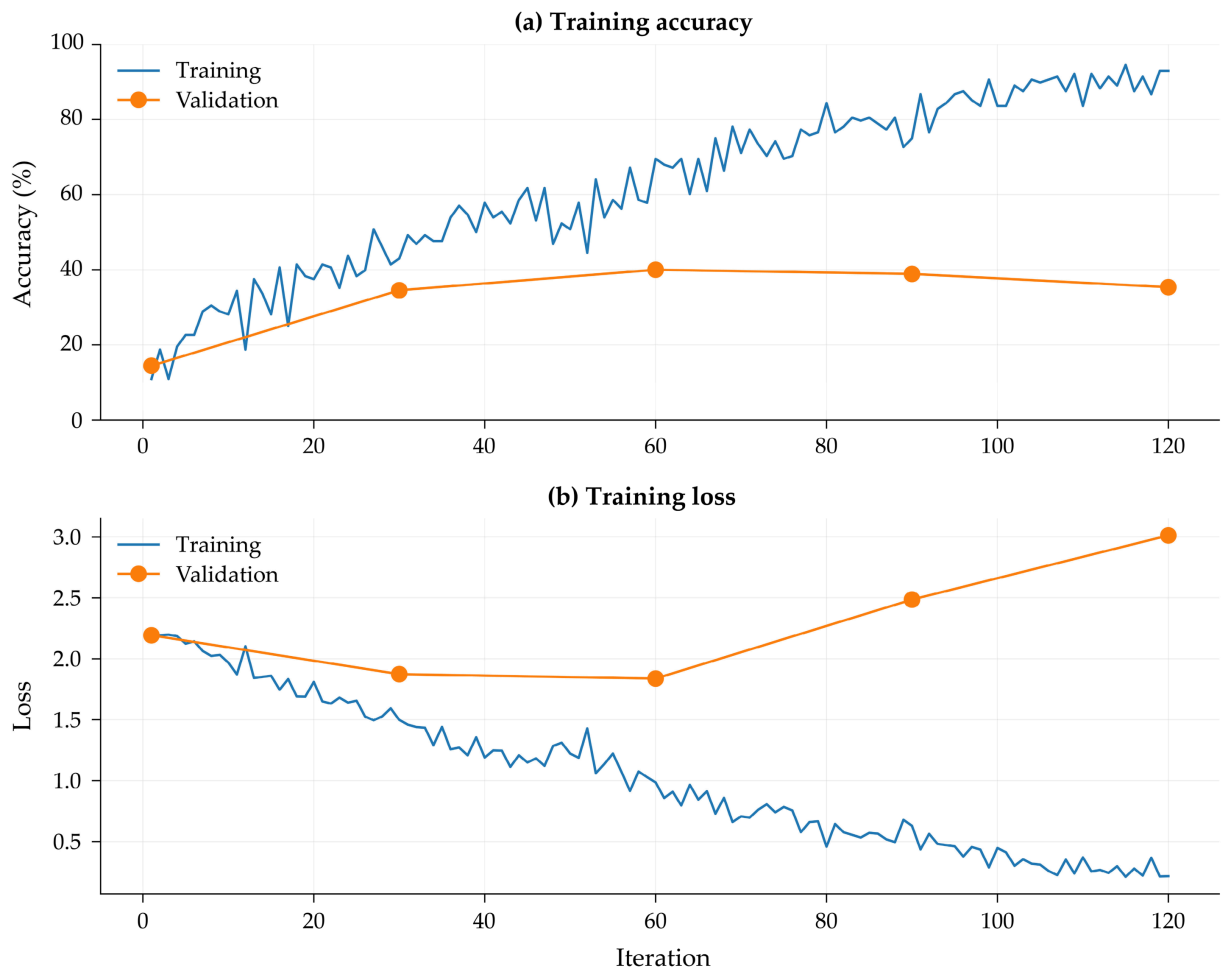
Initial training performance of the CNN: (**a**) training and validation accuracy, (**b**) training and validation loss curves.

Figure 10 presents the initial training performance of the CNN. As shown in Figure 10a, training accuracy increased progressively and exceeded 90%, whereas validation accuracy remained below 40%. This large discrepancy indicates substantial overfitting, suggesting that the model learned patterns specific to the training dataset but exhibited limited generalization capability. Figure 10b further supports this observation, showing a continuous decrease in training loss while validation loss remained high and increased during later training stages.

**Figure 11:**
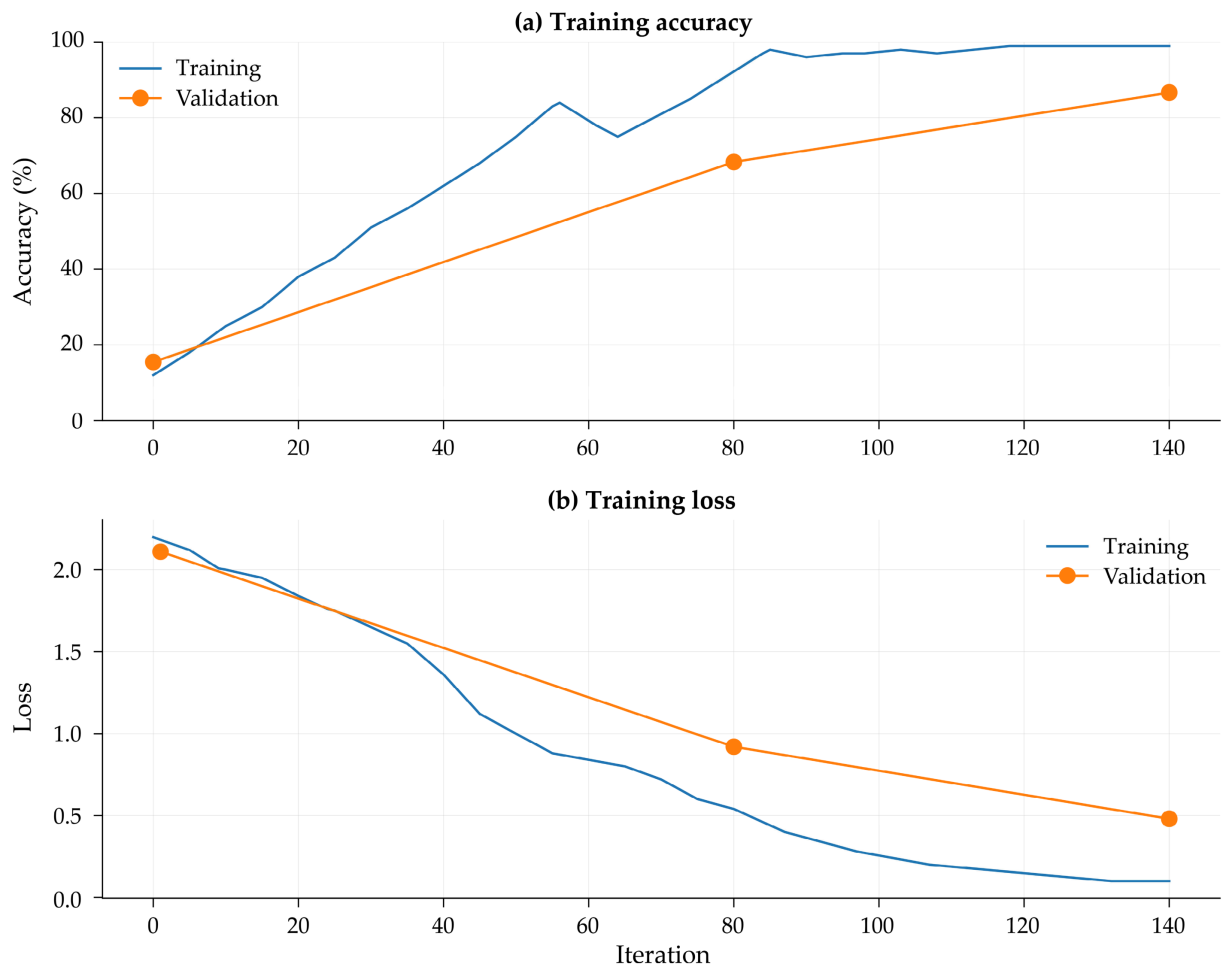
Optimized training performance of the CNN after hyperparameter adjustment: (**a**) training and validation accuracy, (**b**) training and validation loss curves.

Following hyperparameter adjustment, the training dynamics improved considerably, as illustrated in Figure 11. In Figure 11a, validation accuracy increased progressively to 86.67%, reducing the gap relative to training accuracy and indicating a more stable learning process. Similarly, Figure 11b shows concurrent reductions in training and validation losses throughout training. These results demonstrate a substantial improvement in model generalization compared with the initial configuration.

The optimized model achieved higher validation performance while maintaining stable convergence behavior. This improvement provided the basis for the subsequent evaluation of classification performance using the independent test dataset.

### 3.2. Classification Performance and Result Analysis

**Figure 12:**
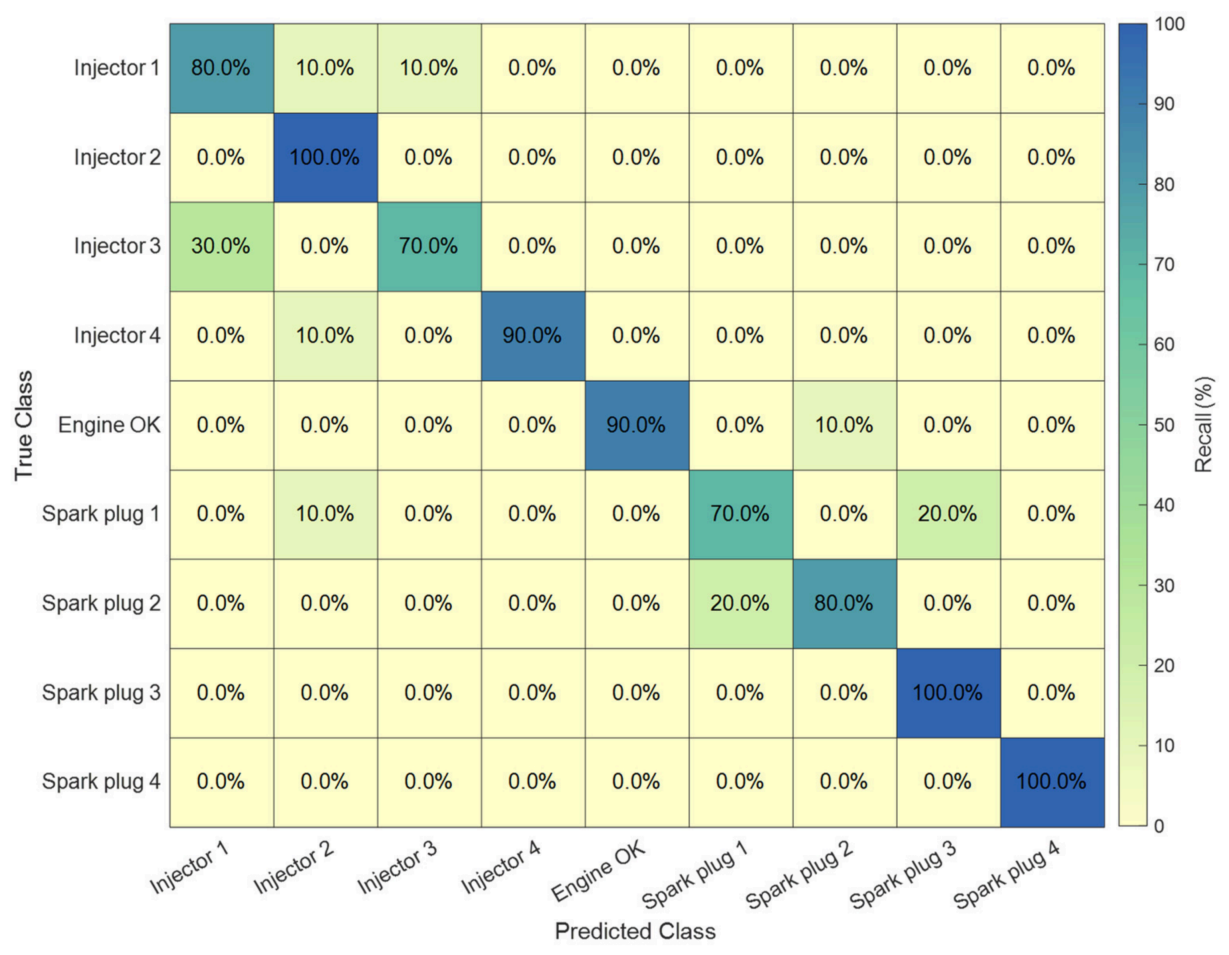
Confusion matrix of the CNN model, normalized by rows (recall %).

The normalized confusion matrix in Figure 12 provides a detailed overview of the CNN’s classification performance under each operating condition. Table 6 complements this analysis by reporting precision, recall, and F1-score values for all classes.

The proposed SDP–CNN framework achieved an overall accuracy of 81.11% on the independent test dataset. However, performance varied among the different operating conditions. The highest classification performance was achieved by Spark Plug 3 and Spark Plug 4, with perfect precision, recall, and F1-score of 1.000. Similarly, Injector 4 and Engine OK exhibited high performance, reaching a recall of 0.900, precision of 1.000, and an F1-score of 0.947.

Injector 2 also demonstrated strong classification performance, achieving a recall of 1.000 and an F1-score of 0.870. Although all samples in this class were correctly identified, a small number of samples from other classes were assigned to Injector 2, resulting in a lower precision of 0.769.

The lowest classification performance was observed for Injector 1, Injector 3, and Spark Plug 1, with F1-scores of 0.762, 0.778, and 0.700, respectively. As shown in Figure 12, Injector 1 was misclassified as Injector 2 and Injector 3 in 10% of the cases each, whereas Injector 3 was misclassified as Injector 1 in 30% of the samples. Similarly, Spark Plug 1 was misclassified as Spark Plug 3 in 20% of the cases and as Injector 2 in 10% of the samples. Spark Plug 2 achieved intermediate performance, with precision, recall, and F1-score values of 0.800.

Overall, the confusion matrix indicates that classification errors were concentrated in a limited number of operating conditions, whereas several classes achieved near-perfect or perfect recognition rates. These results demonstrate the proposed methodology’s ability to differentiate among multiple injector and spark plug fault conditions using SDP-based acoustic representations and convolutional neural network classification.

## 4. Discussion

The results obtained demonstrate that the acoustic methodology based on SDP and CNN achieved competitive performance, reaching an overall accuracy of 81.11% on the independent verification dataset. The best results were obtained for Spark Plug 3, Spark Plug 4, and Injector 4 (F1 ≥ 0.94), whereas Injector 1, Injector 3, and Spark Plug 1 presented greater classification complexity (F1 ≈ 0.70–0.78). During the initial training stages, overfitting was observed, as evidenced by the divergence between the training and validation curves. This behavior was mitigated through hyperparameter tuning, highlighting the importance of proper optimization when working with moderately sized datasets.

These findings are consistent with recent studies highlighting the effectiveness of advanced acoustic representations and deep learning architectures for complex signal classification tasks. The Symmetrized Dot Pattern (SDP) method offers several advantages for such applications, including robustness to background noise, sensitivity to short-duration transient phenomena, and natural compatibility with convolutional neural networks by transforming temporal signals into visual representations [13,16,17]. These characteristics were particularly evident in classes exhibiting well-defined acoustic signatures, where high precision and recall values were achieved.

The lower classification performance observed for Injector 1, Injector 3, and Spark Plug 1 is likely attributable to the intrinsic similarity of their acoustic signatures rather than to random classification errors. In a multicylinder internal combustion engine, combustion events occur sequentially according to the firing order, generating partially overlapping acoustic emissions that propagate through common structural elements such as the cylinder block, cylinder head, and intake and exhaust systems before reaching the microphone. Consequently, faults affecting individual cylinders may produce highly similar spectral energy distributions, particularly when measurements are acquired from a single external microphone position. Since the SDP representation is derived from the magnitude spectrum, operating conditions with comparable spectral distributions may also yield visually similar dot patterns, reducing the discriminative information available to the CNN. This behavior is consistent with the confusion matrix presented in Figure 12, where most misclassifications occurred between classes exhibiting similar acoustic characteristics rather than between fundamentally different fault categories.

The high classification performance achieved in this study reinforces previous evidence indicating that acoustic emissions from internal combustion engines contain diagnostically relevant information that can be captured using MEMS microphone-based acquisition systems and exploited through machine learning techniques [11,12,23]. In this context, the proposed methodology extends these findings by incorporating SDP representations, which transform acoustic signals into structured visual patterns, thereby enabling CNNs to automatically extract discriminative features. This strategy enabled the differentiation of multiple conditions associated with injection and ignition faults in multicylinder engines, an application that remains relatively limited in the available literature.

From a deep learning perspective, the results are consistent with previous investigations that report the potential of convolutional architectures for analyzing complex, high-dimensional signals [8,23]. Nevertheless, the reduction in F1-scores observed for certain classes highlights the challenges associated with generalization when partially overlapping acoustic features are present. This behavior is consistent with observations reported in acoustic engine diagnostics studies, where certain operating conditions generate similar spectral signatures that complicate class separation [12,23]. Furthermore, the presence of residual confusion among some categories suggests that future research could benefit from transfer learning, data augmentation, and domain adaptation strategies, approaches that have demonstrated significant improvements in classification tasks based on acoustic and vibration signals [19,21].

The experiments were intentionally conducted under controlled laboratory conditions to evaluate the intrinsic capability of the proposed SDP–CNN framework while minimizing external acoustic variability. The use of a semi-anechoic chamber, fixed microphone positioning, steady-state engine operation, and deactivation of the cooling fan minimized environmental disturbances, allowing the observed classification performance to be primarily attributed to differences among the induced fault conditions rather than to uncontrolled noise sources. Consequently, the reported accuracy should be interpreted as a proof of concept demonstrating the feasibility of the proposed methodology under controlled laboratory conditions rather than as a direct representation of field performance. Future studies should therefore evaluate the robustness of the proposed framework under progressively more realistic operating conditions, including different engine speeds and loads, multiple microphone locations, varying signal-to-noise ratios, and multiple engine platforms.

Overall, the results demonstrate the feasibility of the SDP–CNN combination for fault detection through acoustic analysis in internal combustion engines. The proposed methodology successfully differentiated conditions associated with injection and ignition faults using non-invasively acquired acoustic signals processed through SDP representations. In addition to extending the application of this technique to multicylinder engines, the study lays the foundation for future research to improve the robustness, generalization, and applicability of deep learning-based acoustic diagnostic systems in real operating environments.

## 5. Conclusions

An acoustic fault detection methodology based on Symmetrized Dot Patterns (SDPs) and convolutional neural networks (CNNs) was developed and evaluated for multicylinder internal combustion engines. Acoustic signals acquired with MEMS microphones were converted into SDP images and classified using a CNN, achieving an overall accuracy of 81.11% on an independent test set under controlled laboratory conditions.

The results demonstrate the feasibility of using SDP representations to preserve discriminative acoustic information associated with injector and spark plug fault conditions, enabling automatic classification through deep learning techniques. Although the proposed methodology was validated under controlled experimental conditions using a single-engine platform, it establishes a proof of concept that extends the application of SDP to multicylinder engine diagnostics. Future research should focus on larger datasets, multiple engine platforms, varying operating conditions, and more realistic acoustic environments to further evaluate the robustness and generalization capability of the proposed approach.

## Figures and Tables

**Table 1:** Technical specifications of the internal combustion engine.

Parameter	Value
Maximum power	136 HP/100 kW
Maximum torque	180 Nm
RPM at maximum power	5800 rpm
RPM at maximum torque	4800 rpm
Number of cylinders	4
Compression ratio	10:1
Displacement	1.997 L
Cylinder arrangement	Inline

**Table 2:** Technical specifications of the smartphone MEMS microphone used for acoustic signal acquisition.

Parameter	Value
Microphone type	MEMS (digital, integrated)
Number of microphones	Three (multi-position array)
Sampling frequency	48 kHz
Frequency response range	20 Hz–20 kHz (nominal)
Sensitivity	~−38 dBV ± 3 dB (typical)
Signal-to-noise ratio (SNR)	~65 dB (estimated)
Acoustic overload point (AOP)	~120 dB SPL (estimated)
Operating system	iOS

**Table 3:** Parameters of combination.

Parameter Combination	Visual Symmetry	Lobe Separation	Pattern Clarity	Accuracy
l = 20, ξ = 15°	Low	Low	Low	73.33%
l = 20, ξ = 30°	Medium	Medium	Medium	75.56%
l = 20, ξ = 45°	Low	Excessive	Low	57.41%
l = 80, ξ = 30°	Excellent	Excellent	Excellent	81.11%
l = 100, ξ = 15°	Good	Medium	Medium	75.56%
l = 100, ξ = 30°	Good	Good	Good	80.00%
l = 100, ξ = 45°	Low	Excessive	Low	62.22%

**Table 4:** Detailed CNN architecture.

Stage	Layer	Kernel Size	Filters/Neurons	Stride	Padding	Output Size
1	Image input	224 × 224 × 3	—	—	—	224 × 224 × 3
2	Convolution + BN + ReLU	6 × 6	16	1	Same	224 × 224 × 16
3	Max pooling	3 × 3	—	3	—	74 × 74 × 16
4	Convolution + BN + ReLU	6 × 6	26	1	Same	74 × 74 × 26
5	Max pooling	2 × 2	—	2	—	37 × 37 × 26
6	Convolution + BN + ReLU	3 × 3	36	1	Same	37 × 37 × 36
7	Convolution + BN + ReLU	6 × 6	46	1	Same	37 × 37 × 46
8	Max pooling	2 × 2	—	2	—	18 × 18 × 46
9	Convolution + BN + ReLU	6 × 6	56	1	Same	18 × 18 × 56
10	Convolution + BN + ReLU	6 × 6	66	1	Same	18 × 18 × 66
11	Convolution + BN + ReLU	6 × 6	76	1	Same	18 × 18 × 76
12	Max pooling	3 × 3	—	3	—	6 × 6 × 76
13	Convolution + BN + ReLU	6 × 6	86	1	Same	6 × 6 × 86
14	Max pooling	2 × 2	—	2	—	3 × 3 × 86
15	Convolution + BN + ReLU	6 × 6	96	1	Same	3 × 3 × 96
16	Convolution + BN + ReLU	6 × 6	106	1	Same	3 × 3 × 106
17	Max pooling	2 × 2	—	2	—	1 × 1 × 106
18	Convolution + BN + ReLU	6 × 6	116	1	Same	1 × 1 × 116
19	Convolution + BN + ReLU	6 × 6	126	1	Same	1 × 1 × 126
20	Fully connected + ReLU	—	256	—	—	256
21	Fully connected + ReLU	—	128	—	—	128
22	Fully connected + ReLU	—	64	—	—	64
23	Fully connected + ReLU	—	32	—	—	32
24	Fully connected	—	9	—	—	9
25	Softmax	—	9 classes	—	—	9 classes
26	Classification	Cross-entropy	—	—	—	—

**Table 5:** Training hyperparameters.

Hyperparameter	Value
Software	MATLAB R2024a
Framework	Deep Learning Toolbox
Optimizer	Adam
Initial learning rate	0.001
Maximum epochs	60
Input image size	224 × 224 × 3
Activation function	ReLU
Batch normalization	After every convolutional layer
Loss function	Categorical cross-entropy
Validation frequency	Every 80 iterations
Shuffle	Every epoch
Dropout	Not used
L2 regularization	Not used
Number of output classes	9

**Table 6:** Precision, recall, and F1-score of the CNN for each engine condition.

Class	Precision	Recall	F1-Score
Engine OK	1.000	0.900	0.947
Injector 1	0.727	0.800	0.762
Injector 2	0.769	1.000	0.870
Injector 3	0.875	0.700	0.778
Injector 4	1.000	0.900	0.947
Spark plug 1	0.700	0.700	0.700
Spark plug 2	0.800	0.800	0.800
Spark plug 3	1.000	1.000	1.000
Spark plug 4	1.000	1.000	1.000

## Data Availability

Data are contained within the article.

## Abbreviations

The following abbreviations are used in this manuscript:

ICE	Internal Combustion Engine
SDP	Symmetrized Dot Pattern
CNN	Convolutional Neural Network
DFT	Discrete Fourier Transform
FFT	Fast Fourier Transform
MEMS	Micro-Electro-Mechanical Systems
OBD-II	On-Board Diagnostics II
SNR	Signal-to-Noise Ratio
ReLU	Rectified Linear Unit
